# In the December 2011 issue

**DOI:** 10.1590/S1980-57642011DN05040001

**Published:** 2011

**Authors:** Ricardo Nitrini

**Affiliations:** Editor-in-Chief

In this issue we are publishing eight reviews, four original papers and brief reviews. In the September 2011 issue we started the publications of the Recommendations of the Scientific Department of Cognitive Neurology and Aging of the Brazilian Academy of Neurology. We continue to publish these recommendations, now focusing on those related to vascular cognitive impairment and to the differential diagnosis between dementia and other psychiatric conditions.

In the opening session of the 7^th^ Congress on Brain, Behavior and Emotions, **Gianetti**, a philosopher, delivered a lecture on the brain-mind puzzle discussed from the perspective of physicalism, which regards mental events as epiphenomena, without causal influence on human's behavior. We are very grateful for his acceptance to publish this highly intriguing lecture in our journal.

**Engelhardt et al.** present the recommendations of the Brazilian Academy of Neurology for the diagnosis of vascular dementia and for vascular cognitive impairment without dementia together with the suggested supplementary exams. This is relevant issue for a condition that may be more frequent in developing countries

**Engelhardt et al.** also present the recommendations of the Brazilian Academy of Neurology for the cognitive, functional and behavioral assessment in vascular cognitive impairment and dementia. Tests, scales and questionnaires that have been adapted and/or validated for using in Brazil, especially those taking into account both schooling and age, are highlighted.

**Brucki et al.** review the treatment of vascular dementia and present the recommendations of the Brazilian Academy of Neurology for this frequent and challenging condition using an evidence-based analysis. There are conclusive studies supporting the recommendations of regular physical activity, adequate diet and control of arterial hypertension, among others, for the treatment (and prevention) of vascular cognitive impairment.

**Bottino et al.** present the recommendations of the Brazilian Academy of Neurology for the differential diagnosis between psychiatric disorders (mainly depression, *delirium*, and use of psychoactive substances) and dementia, with special focus on the dementia of Alzheimer's disease (AD) and vascular dementia (VD).

**Capucho and Brucki** review research studies on judgment, which is described as "the capacity of making decisions after consideration about available information, contextual factors, possible solutions and probable outcomes". The authors found that there is a lack of instruments for evaluating judgment and that data on judgement in mild cognitive impairment and dementia are scarce.

**Abrahão et al.** review the current data on multiple system atrophy, a condition in which cognitive impairment had not been considered a clinical feature until recently. Comprehensive neuropsychological assessment have been able to detect dementia in as much as 14-16% of the cases.

**Sanches et al.** present the cross-cultural adaptation of the Funtional Activities Questionnaire - FAQ for use in Brazil. A previous version of this questionnaire, proposed and used by a World Health Organization study and translated from the Spanish, has been widely used in Brazil. This new version is welcome and a comparison between the old and new version is now needed.

**Miranda et al.** investigated the causes of delay in the diagnosis of Alzheimer's disease in a public university hospital. Educational levels of the caregivers, as well as lack of knowledge of dementia by the members of the family, are relevant factors for the delay in diagnosis.

**Oliveira et al.** performed a systematic review of the neurobiological aspects of memory in the aging process trying to highlight the neural networks and neurotransmitters that are more involved in processing information. The memory changes and the brain morphological alterations in aging are also reviewed.

**Santos et al.** compared the effects of oral and transdermal rivastigmine on cholinesterase inhibitor levels in the serum of patients with Alzheimer's disease. Although there were no clinical or cognitive differences, the authors found that transdermal rivastigmine significantly reduced serum butyryl-cholinesterase levels in the AD.

**Zimmermann et al.** evaluated pragmatic and executive functions in right brain damaged and in traumatic brain injured patients looking for possible dissociations between these functions.

**Ricardo Nitrini** Editor-in-Chief

